# Squamous cell transformation as a mechanism of acquired resistance to tyrosine kinase inhibitor in EGFR‐mutated lung adenocarcinoma: a report of two cases

**DOI:** 10.1002/rcr2.521

**Published:** 2020-01-14

**Authors:** Hironori Uruga, Takeshi Fujii, Nobuyuki Nakamura, Shuhei Moriguchi, Kazuma Kishi, Hisashi Takaya

**Affiliations:** ^1^ Department of Respiratory Medicine, Respiratory Center Toranomon Hospital Tokyo Japan; ^2^ Department of Respiratory Medicine Toranomon Hospital Kajigaya Kawasaki Japan; ^3^ Okinaka Memorial Institute for Medical Research Tokyo Japan; ^4^ Department of Pathology Toranomon Hospital Kajigaya Kawasaki Japan; ^5^ Department of Pathology and Clinical Laboratories National Cancer Center Hospital East Kashiwa Japan; ^6^ Department of Respiratory Medicine Toho University Graduate School of Medicine Tokyo Japan

**Keywords:** Epidermal growth factor receptor‐tyrosine kinase inhibitor, non‐small cell lung cancer, *PI3K/AKT/mTOR*, *PTEN*, squamous cell transformation

## Abstract

Pathological transformation to squamous cell carcinoma after epidermal growth factor receptor (*EGFR*)‐tyrosine kinase inhibitor treatment has been reported, but details of the transformation remain unclear. We report two cases with transformation to squamous cell carcinoma. The first case was a 61‐year‐old man who was an ex‐smoker with stage IV lung adenocarcinoma harbouring *EGFR* exon 19 insertion. He experienced squamous cell transformation after 28 months of erlotinib therapy. Next‐generation sequencing (NGS) analysis showed *EGFR* T790M and genomic alterations in *PTEN*, *PDGFR*, and *HRAS*. The second case was a 72‐year‐old man who was an ex‐smoker with stage IV lung adenocarcinoma harbouring *EGFR* exon 21 L858R. He experienced squamous cell transformation after nine months of erlotinib therapy. NGS analysis showed *EGFR* T790M and genomic alterations in *PTEN*, *SMARCB1*, *TP53*, and *KIT*. Both patients had *PTEN* genomic alterations and the PI3K/AKT/mTOR (mammalian target of rapamycin) pathway might play an important role in squamous cell transformation.

## Graphical abstract

Pathological transformation to squamous cell carcinoma after epidermal growth factor receptor (EGFR)‐tyrosine kinase inhibitor (TKI) treatment has been reported, but details of the transformation remain unclear. Here, we report two cases with transformation to squamous cell carcinoma after treatment with EGFR‐TKIs. Both patients had PTEN genomic alterations and the PI3K/AKT/mTOR (mammalian target of rapamycin) pathway might play an important role in squamous cell transformation.

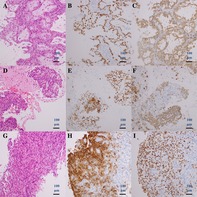

## Introduction

Chemotherapy‐naïve patients with epidermal growth factor receptor (*EGFR*) mutations show a high response rate of 60–70% to first‐ and second‐generation EGFR‐tyrosine kinase inhibitors (TKIs) 1. However, almost all patients treated with EGFR‐TKIs subsequently develop EGFR‐TKI resistance. The most common mechanism of resistance to first‐ and second‐generation EGFR‐TKIs is the T790M mutation, accounting for 60% of patients 2, 3. *EGFR* and mesenchymal–epithelial transition proto‐oncogene amplifications and phosphatidylinositol‐4,5‐bisphosphate 3‐kinase catalytic subunit alpha gene (*PIK3CA*) mutations have also been reported as molecular resistance mechanisms 2. Pathological transformation after EGFR‐TKI treatment has not been adequately studied, but transformation to small cell carcinoma and epithelial–mesenchymal transition are the most common 2, 4. Although pathological transformation to squamous cell carcinoma has been described in some case reports 5, 6, 7, 8, 9, 10, the details have not been elucidated. Here, we report two cases with transformation to squamous cell carcinoma after treatment with EGFR‐TKIs.

## Case Report

### Case 1

**Figure 1:**
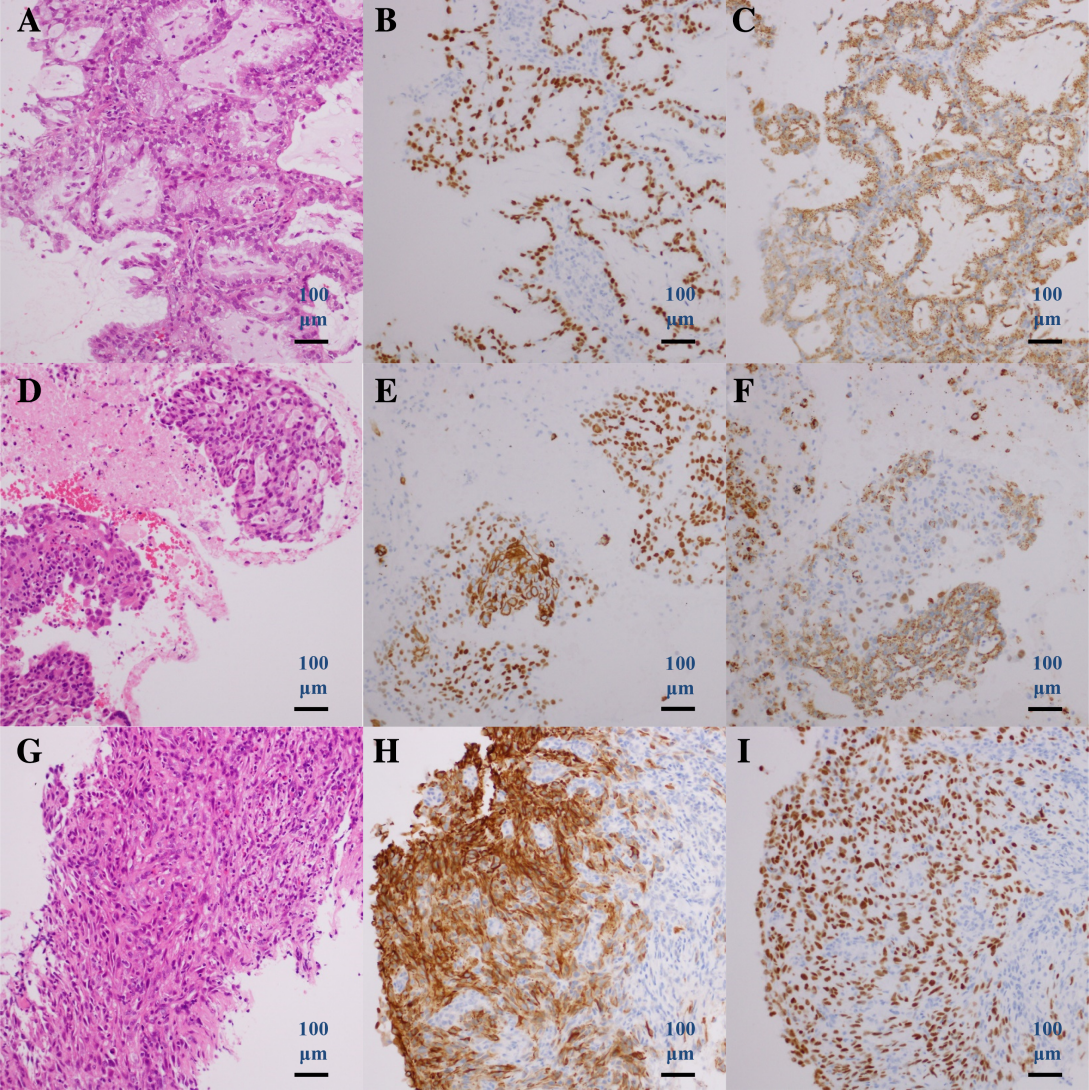
Case 1. Histology of computed tomography‐guided percutaneous lung biopsy specimen before erlotinib therapy (A–C). (A) Haematoxylin and eosin, (B) TTF‐1 and CK5/6, and (C) p40 and napsin A staining. Histology of transbronchial lung biopsy specimen after erlotinib therapy, suggesting squamous cell transformation (D–F). (D) Haematoxylin and eosin, (E) TTF‐1 and CK5/6, and (F) p40 and napsin A staining. Histology of transbronchial lung biopsy specimen after combination chemotherapy (G–I). (G) Haematoxylin and eosin, (H) TTF‐1 and CK5/6, and (I) p40 and napsin A staining.

A 61‐year‐old man who was an ex‐smoker underwent computed tomography‐guided percutaneous lung biopsy. On the basis of the American Joint Committee on Cancer staging system, seventh edition, he was diagnosed as having stage IV (T2bN2M1a) adenocarcinoma of the lung (Fig. 1A–1C) harbouring *EGFR* exon 19 insertion without T790M mutation. He received erlotinib (150 mg daily) and showed a partial response. After 28 months of therapy, the primary lung lesion started to advance. Transbronchial lung biopsy of the lesion showed adenosquamous carcinoma harbouring EGFR exon 19 insertion without *EGFR* T790M mutation (Fig. 1D, E). He received four cycles of combination chemotherapy with immune checkpoint inhibitor plus carboplatin (area under the concentration–time curve 5 on day 1 and every three weeks), paclitaxel (200 mg/m^2^ on day 1 and every three weeks), and atezolizumab (1200 mg on day 1 and every three weeks), then following maintenance therapy of atezolizumab (1200 mg every three weeks), and showed a partial response. After 14 months of therapy, the primary lung lesion worsened, and new lesions developed with bone metastases to the spine. Transbronchial lung biopsy of the lesion showed squamous cell carcinoma (Fig. 1F, G). Next‐generation sequencing (NGS) analysis of the specimen with Ion AmpliSeq Cancer Hotspot Panel version 2 (Thermo Fisher Scientific, USA) showed *EGFR* c.2369C>T (p.T790M), *PTEN* c.963 del (p.N323Mfs*21), c.964_964 delA (p.N323Mfs*21), c.968 del (p.N323Mfs*21), *PDGFRA* c.2472C>T (p.V824V), and *HRAS* c.81T>C (p.H27H). PTEN expression was assessed immunohistochemically using the *H*‐score 11, and a score of 50 or higher was judged as positive. *H*‐score of PTEN expression was negative both before and after erlotinib therapy. Osimertinib (80 mg daily) was started and achieved stable disease over three months. Following disease progression with osimertinib, the patient was started on pembrolizumab (200 mg on day 1 and every three weeks) but after the first administration, the disease continued to progress. Then, S‐1 (100 mg/kg body weight on days 1–14 and every three weeks) was administered over three months. However, the patient died of lung cancer 58 months after diagnosis, and 17 months after transformation to squamous cell carcinoma.

### Case 2

**Figure 2:**
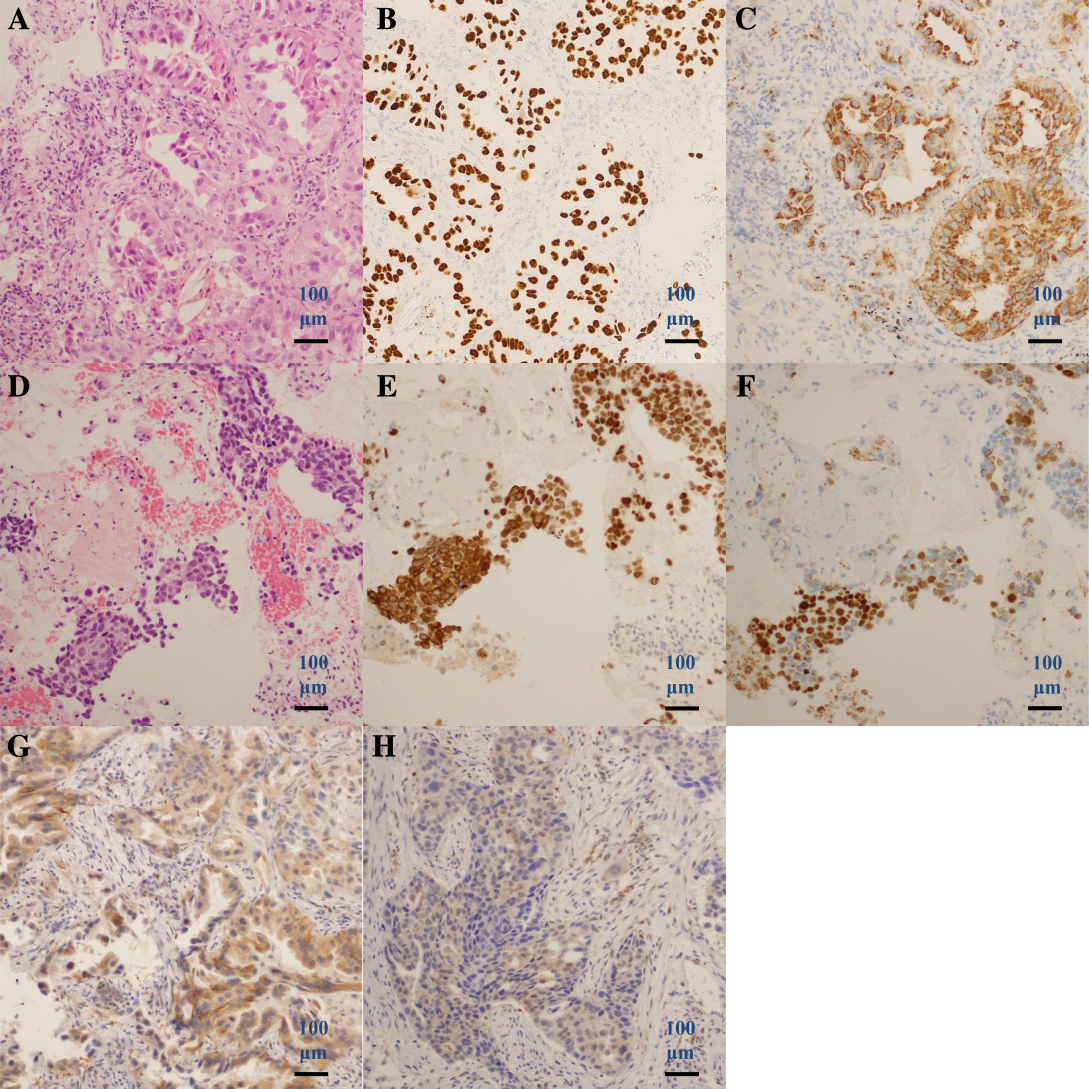
Case 2. Histology of transbronchial lung biopsy specimen before erlotinib therapy (A–C). (A) Haematoxylin and eosin, (B) TTF‐1 and CK5/6, and (C) p40 and napsin A staining. Histology of transbronchial lung biopsy specimen after erlotinib therapy, suggesting squamous cell transformation (D–F). (d) Haematoxylin and eosin, (E) TTF‐1 and CK5/6, and (F) p40 and napsin A staining. PTEN staining before erlotinib therapy (G) and after erlotinib therapy (H).

A 72‐year‐old man who was an ex‐smoker underwent transbronchial lung biopsy. He was diagnosed as having stage IV (T2bN2M1a) adenocarcinoma of the lung (Fig. 2A–2C) harbouring *EGFR* exon 21 L858R without T790M mutation. He received erlotinib (150 mg daily) and showed a partial response. After nine months of this therapy, the primary lung lesion started to advance. Transbronchial lung biopsy of the lesion showed adenosquamous carcinoma (Fig. 2D–2F). NGS analysis of the specimen using Ion AmpliSeq Cancer Hotspot Panel showed *EGFR* c.2573T>G (p.L858R), c.2369C>T (p.T790M), *PTEN* c.963 del (p.N323Mfs*21), c.964_964 delA (p.N323Mfs*21), c.968 del (p.N323Mfs*21), *SMARCB1* c.1119‐41G>A (unknown), *TP53* c.892G>T (p.E298*), and *KIT* c.1621A>C (p.M541L). *H*‐score of PTEN expression had converted from positive to negative after erlotinib therapy (Fig. 2G, H). Osimertinib (80 mg daily) was then started and achieved stable disease over three months. Following disease progression with osimertinib, he could not receive cytotoxic chemotherapy because of poor performance status; thus, he was started on pembrolizumab (200 mg on day 1 and every three weeks) but showed disease progression after the first administration. The patient died of lung cancer 31 months after diagnosis, and eight months after transformation to squamous cell carcinoma.

## Discussion

We encountered two patients with pathological transformation to squamous cell carcinoma as a mechanism of resistance to EGFR inhibitors. NGS analysis of both specimens after erlotinib therapy showed genomic alterations in *PTEN*.

Squamous cell transformation as a resistance mechanism to EGFR inhibitors has been investigated clinically. Roca et al. 12 performed a pooled analysis of 17 patients and showed that most of these patients were smokers and harboured original *EGFR* mutations. In addition to squamous cell transformation, they observed coexisting *EGFR* T790M mutation in approximately half of the patients. Prognosis after squamous cell transformation was poor, with a median overall survival of 3.5 months. In our two patients, osimertinib was administered because of the coexisting *EGFR* T790M mutation in specimens after erlotinib therapy. However, we achieved control of the lung cancer with squamous cell transformation and *EGFR* T790M by osimertinib for only three months in both patients. AURA3 was a phase 3 study comparing osimertinib and platinum‐pemetrexed in patients with *EGFR* T790M‐positive non‐small cell lung cancer after erlotinib therapy or gefitinib therapy. In this study, patients with squamous cell histology were three of 279 (1%) in osimertinib group, and zero of 140 (0%) in platinum plus pemetrexed group. The study showed that osimertinib resulted in significantly better progression‐free survival (PFS) than platinum‐pemetrexed (10.1 vs. four months, respectively, *P* < 0.001) 13. This was better than the PFS of three months in our patients with squamous cell transformation and *EGFR* T790M mutation. Thus, the prognosis of lung cancer patients with squamous cell transformation and *EGFR* T790M mutation appears to be worse than that of patients with only *EGFR* T790M mutation. This is consistent with a report by Roca et al. 12.

At the molecular level, *PTEN* genomic alterations were identified in our patients with squamous cell transformation. In a study of surgically resected specimens, *PTEN* mutations were more often identified in ex‐smokers and in squamous cell carcinomas than in adenocarcinomas 14. Park et al. 15 performed NGS analysis of specimens before and after squamous cell transformation following EGFR‐TKI therapy, and showed genomic alterations in *PTEN* and *PIK3CA* in each two of four patients. Another case report by Kuiper et al. 5 also showed genomic alteration of *PIK3CA* in a specimen after squamous cell transformation. Park et al. 15 hypothesized that the *PI3K/AKT/mTOR* pathway was activated by EGFR‐TKIs and loss of *PTEN*, which facilitates cell proliferation and resulted in squamous cell transformation. Interestingly, they found that one of four patients received everolimus (an mTOR (mammalian target of rapamycin) inhibitor) and showed radiological improvement. Further studies and more case reports are warranted to clarify the clinical utility of mTOR inhibitors in *EGFR*‐mutated patients with squamous cell transformation.

The present report has several limitations. First, the primary lung cancer before treatment with erlotinib therapy could have been adenosquamous cell carcinoma ab initio. We could not rule out this possibility because both patients were diagnosed using biopsy specimens. Second, we could not perform NGS analysis of the specimens before treatment with erlotinib, because the specimen volume was inadequate for analysis.

In summary, we encountered two cases of *EGFR* mutation in lung adenocarcinoma with pathological transformation to squamous cell carcinoma. NGS analysis showed *PTEN* genomic alterations in both cases. Osimertinib was not fully effective in patients with squamous cell transformation, thus cytotoxic chemotherapies were probably better for these patients. Further studies and more case reports are warranted to elucidate the underlying mechanisms and investigate treatment modalities for patients with squamous cell transformation.

### Disclosure Statement

Appropriate written informed consent was obtained for publication of this case report and accompanying images.
